# New developments in surgery of malignant gliomas

**DOI:** 10.2478/v10019-011-0018-3

**Published:** 2011-07-20

**Authors:** Andrej Vranic

**Affiliations:** Department of Neurosurgery, UMC Ljubljana, Slovenia

**Keywords:** malignant gliomas, surgery, neuronavigation, image-guided surgery, transcranial magnetic stimulation, fluorescence-guided resection

## Abstract

**Background:**

Malignant gliomas account for a high proportion of brain tumours. With new advances in neurooncology, the recurrence-free survival of patients with malignant gliomas has been substantially prolonged. It, however, remains dependent on the thoroughness of the surgical resection. The maximal tumour resection without additional postoperative deficit is the goal of surgery on patients with malignant gliomas. In order to minimize postoperative deficit, several pre- and intraoperative techniques have been developed.

**Conclusions:**

Several techniques used in malignant glioma surgery have been developed, including microsurgery, neuroendoscopy, stereotactic biopsy and brachytherapy. Imaging and functional techniques allowing for safer tumour resection have a special value. Imaging techniques allow for better preoperative visualization and choice of the approach, while functional techniques help us locate eloquent regions of the brain.

## Introduction

Recent advances in various disciplines have delineated the molecular basis of brain tumours and introduced new technologies in oncology and immunology. New chemotherapeutic agents with few systemic adverse effects have become available, offering hope of better treatment options for patients with malignant gliomas.^1^,^2^ These agents work by inhibiting tumour growth, angiogenesis, proliferation, invasion and spread of the tumour. Monoclonal antibodies, either alone or carrying a cytotoxic payload, promise to control the tumour growth. Gene therapy is maturing, with several clinical trials under way in which improved vectors and new therapeutic genes are being used to target tumour cells. Additional therapeutic approaches include the use of radiation sensitizers, optimization of current radiation modalities, electrophysiological interference with cell proliferation and stem cell-based approaches.^2^

In spite of all these advances, the survival of patients with malignant gliomas is still closely correlated with the more extensive tumour resection.^3^,^4^ Even when the radical removal of a brain tumour is not possible, surgery provides the diagnosis and prevents symptoms of mass effect. Tissue obtained during surgery is critical to allow for the individualized approach in designing treatments with newer therapies that are becoming available.

In recent years, several new techniques facilitating malignant brain tumour surgery have become available. Technical advances help us to minimize the injury to the surrounding healthy brain tissue and the consequent postoperative neurological deficit. This is especially important in case of tumours, growing in or bordering to the *eloquent regions* of the brain. In narrow terms, eloquent regions are regions enabling the fluent speech (*lat. eloquens* = fluent). In broader meaning, eloquent regions are all cortical regions essential for human integrity – speech, motor, visual and sensory areas. While removing brain tumours or obtaining brain tumours tissue for diagnosis, damage of eloquent regions during brain surgery should be avoided by any cost.

## Microsurgery

The microsurgical resection remains the basic technique in neurosurgery and the most important therapeutic modality in the management of malignant gliomas. There has never been a controlled, randomized study to determine whether simple debulking of tumours is as effective as maximal cytoreduction. However, evidence suggests that the more extensive surgical resection is associated with longer life expectancy for patients with high-grade gliomas.^3^,^4^ Next to the operating microscope, basic microsurgical tools are the bipolar forceps, micro aspirator and other micro instruments. *CUSA (Cavitron Ultra Sonic Aspirator)* can be useful when removing tumours that are less vascularized. Precise knowing of brain anatomy structures is of paramount importance and no technique can replace surgeon’s microanatomical knowledge. However, anatomic and functional variability of cortical gyri and sulci can make surgery in the cerebral hemispheres difficult. The exact spatial location of a deep-seated intracranial lesion is often difficult to define on the basis of two-dimensional CT and MR images.^5^

Several pre- and intraoperative techniques are available to make the microsurgical resection of brain tumours more feasible. Most of the techniques are imaging techniques helping us to visualize the tumour before or during surgery. Others are functional techniques enabling us to determine where the eloquent brain areas are located (Table 1).

## Three-dimensional (3D) planning

The problem of the exact spatial localization of the tumour on the basis of two-dimensional MR images has always posed a problem for neurosurgeons. It has been partially overcome by the introduction of computer programs for 3D visualization of medical images. Preoperative 3D visualization of medical images allows a neurosurgeon to perform interactive preoperative 3D planning. The virtual reality environment in which the surgeon reaches with both hands into a computer-generated stereoscopic 3D space can be created.^6^ Surgical targets can be defined and the most suitable surgical approach to the lesion can be selected. In addition, 3D visualization can be employed intraoperatively to locate planned targets by visually matching the computer generated 3D surfaces with the intraoperative view.^5^ 3D visualization proves to be adequate and accurate for locating superficial brain tumours in cases where transfer of planned surgical targets to the surgical field is possible.^5^

## Neuronavigation

**FIGURE 1:**
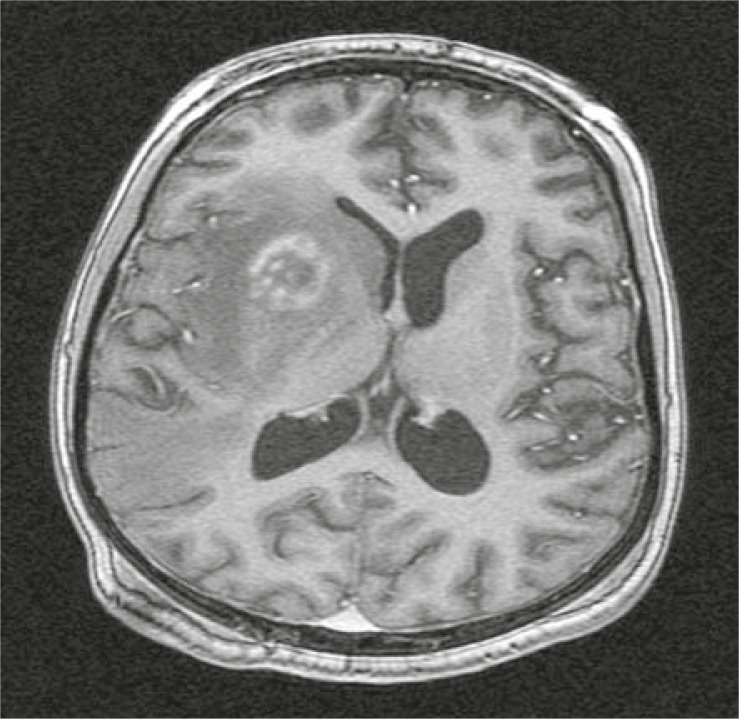
Thalamic glioma eligible for neuronavigation-guided needle biopsy.

At the end of the 20^th^ century, neurosurgery has entered in the era of image guided surgery or neuronavigation.^7^ Frameless, image-guided neuronavigation system is based on MRI scans taken preoperatively and is thus not considered a real-time intraoperative imaging procedure. However, it can provide the surgeon with interactive, dynamic feedback during surgery. The technology uses anatomical points on the patient’s face or adhesive markers attached to the patient’s head as reference points. The working station fuses the position of reference points with preoperative MR scans. The position of the hend-held pointer relatively to the lesion is shown on the screen. With the help of neuronavigation, borders of brain lesions can be easily determined at the beginning of the surgery. Craniotomy and dural incision can be sized accordingly. Craniotomy can be made smaller and more precise owing to the accuracy afforded by image guidance.^7^ Brain shift after craniotomy can disrupt the accuracy and must be accounted for. Neuronavigation is especially suitable for detecting small intraparenchymal lesions where cortex of the brain is expected to be normal. It enables the exact spatial location of small deep-seated lesions before and during the surgical procedure, with about 2 mm accuracy. Biopsy of lesions as small as 0.5 cm in diameter can be safely and successfully performed with the help of neuronavigation-guided biopsy needle (Figure 1).

## Intraoperative ultrasound (iUS)

Improved orientation and visualization of the tumour, adjacent ventricles and peritumoural vasculature is one of the main advantages of the ultrasonography-assisted microsurgery. Its usefulness is most obvious in subcortical cystic gliomas surgery.^8^ Intra-operative ultrasound can be successfully integrated into the neuronavigation system thus offering helpful real-time images of brain tumours.^8^

## Fluorescence–guided resection of malignant gliomas

The use of fluorescent tumour marker for intraoperative detection brain tumours has been shown to enhance the macroscopic total resection of malignant gliomas.^9^ The technique involves oral administration of the nonfluorescent prodrug, 5-aminole-vulinic acid (ALA). In tumour tissue, 5-ALA is metabolized to fluorescent protoporphyrin IX (PpIX) through the heme biosynthesis pathway. PpIX is accumulated in WHO grade III and IV malignant gliomas. Explanations for the higher 5-ALA uptake into the tumour tissue include disrupted BBB, increased neovascularization, and overexpression of membrane transporters in glioma tissue.^10^ The altered pattern of expression of enzymes involved in haemoglobin biosynthesis in tumour cells may also be involved. PpIX levels in normal brain tissue are very low, creating a high tumour-to-normal tissue contrast. The use of a specially adapted operating microscope omitting blue light with wavelength of 400 nm allows the surgeon to visualize brain tissue as “blue” and the tumour as “red” in colour (Figure 2). The intraoperative tumour resection is thereby optimized. A phase 3 study reported a longer recurrence-free survival of patients with malignant gliomas who underwent the fluorescence-guided tumour resection.^9^ Minimal side effects have been reported, including skin photo-sensitivity after the oral administration. Currently, ALA is not yet approved by the FDA in the United States for the surgical resection of brain tumours.^10^ Efforts are underway to perform a controlled, randomized, multicentric trial using fluorescence-guided surgery to determine an effect on the extent of resection.

## Intraoperative MRI (iMRI)

Next to the iUS and fluorescence-guided surgery, iMRI is the only real-time intraoperative technique for visualizing malignant gliomas. It allows surgeons to take MR scans during surgery, while the patient is still in the operating room. Surgery can be temporarily stopped, MRI is performed and MR scans are analysed to determine if the tumour has been removed completely, or if the surgery should continue. Decisions based on current information can be made within minutes. A specially equipped operation theatre is needed, with no ferromagnetic material built into the operation table, surgery tools or the anesthetic equipment. The biggest advantage of the use of the iMRI is that it can help the surgeon to identify the normal tissue in eloquent areas. Although this costly procedure has been on market for more than a decade, only few centres in the world have been able to afford it. Presently around 100 centres worldwide have been equipped with this state-of-art technology.

## Fiber tracking

**FIGURE 3:**
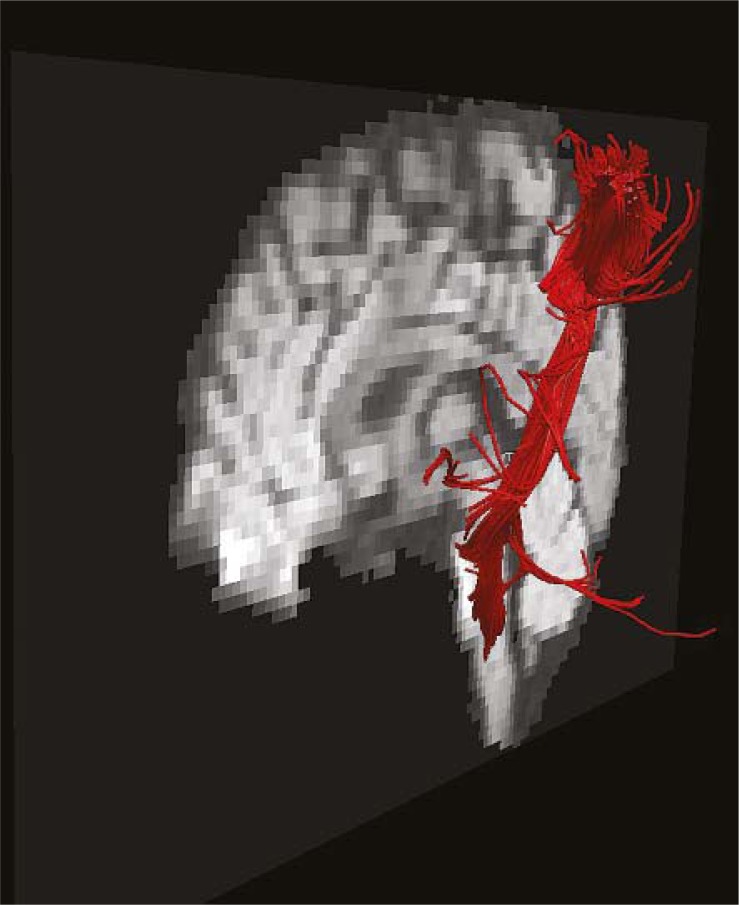
DTI of the corticospinal tract. Kindly provided by Dr. Blaž Koritnik, Institute of Neurophysiology, UMC Ljubljana.

Diffusion tensor imaging (DTI) provides information about eloquent white matter structures. Long tracts, especially the corticospinal tract, can be visualized by means of a technique called *DTI fiber tracking* (Figure 3). Fiber tracking can be integrated into neurosurgical planning software and be used for navigated surgery. During surgery, the corticospinal tract should be avoided.

## Functional MRI (fMRI)

**FIGURE 4:**
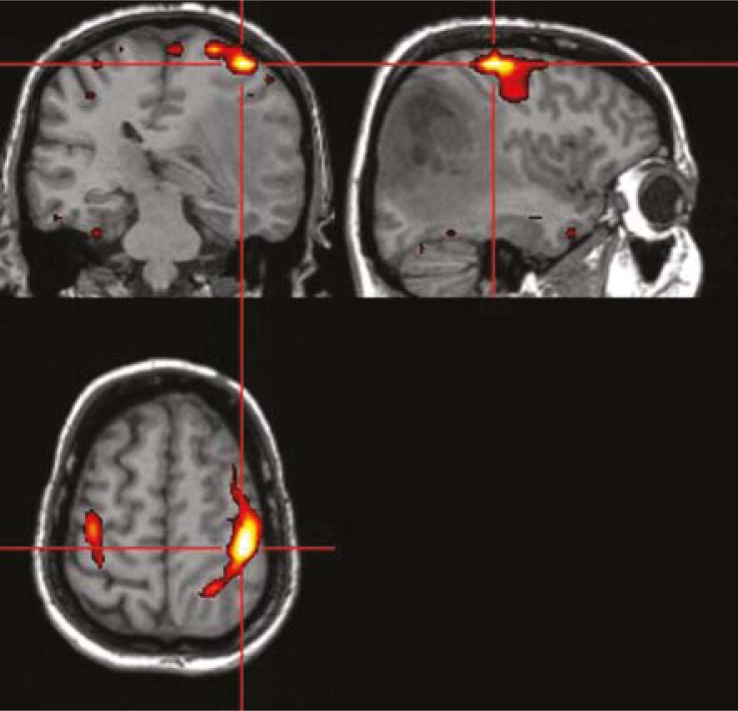
fMRI scan showing primary motor cortex on the right side, anteriorly to a malignant glioma. Kindly provided by Dr. Blaž Koritnik, Institute of Neurophysiology, UMC Ljubljana.

With the help of the fMRI, active parts of the brain are visualized. Higher blood flow through the active regions of the brain is registered while the patient is talking or moving his limbs. MRI scan shows active areas of the brain in different colour (Figure 4). The drawback of this imaging technique is its insufficient accuracy. However, gross impression of the lesion can be obtained by studying fMRI scans preoperatively. Accordingly, eloquent regions can be avoided during surgery.

## Transcranial magnetic stimulation (TMS)

**FIGURE 5:**
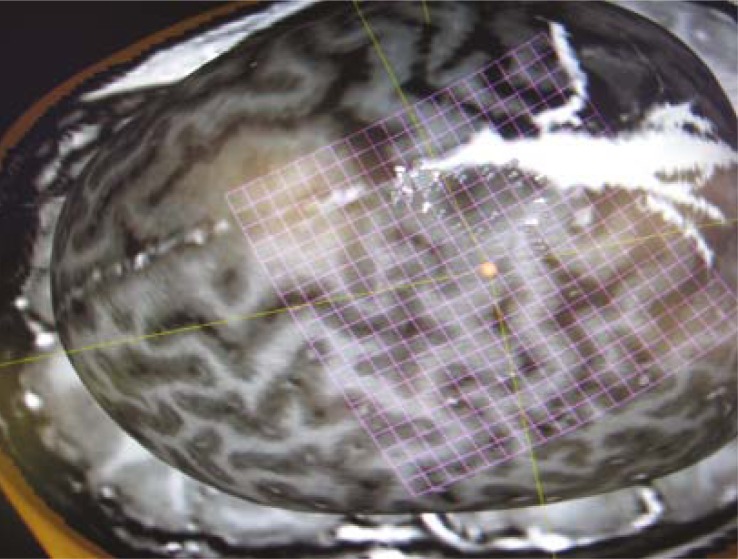
TMS of brain cortex around a glioma of the primary motor cortex.

TMS is a noninvasive method for analysing the cortical function. Its main use is in preoperative functional mapping of the primary motor cortex in parietal tumour surgery. With the help of TMS, the location of primary motor cortex can be determined before surgery, with the patient awake. The manually guided brain stimulator is moved around the patient’s head and small strength magnetic impulses are generated, stimulating brain areas. When primary motor cortex is stimulated, a response on patient’s limbs is recorded by means of electromyography (EMG). *Navigated TMS* fuses the principles of TMS, EMG and neuronavigation.^11^ It allows us to see on the MRI scans exactly where in the cortex the TMS stimulus is given. Exact points of the stimulation are recorded on MR scans in the computer program. With the help of neuronavigation, these images can be used pre- or intraoperatively to enhance the safe microsurgical resection of motor cortex tumours (Figure 5).

## Direct cortical stimulation

Alternatively to TMS, the location of primary motor areas can be determined intraoperatively, with the help of the direct cortical stimulation. Bi- or unipolar electrode is used manually, touching the surface of the brain. Stimulation parameters must be adjusted according to the stimulated area. Motor responses are recorded on the limbs as motor evoked potentials, while the patient is only mildly relaxed. *Brain mapping* is performed as the areas leading to a motor response in the limbs are marked with sterile paper markers and avoided during the tumour removal.

## Awake surgery

**FIGURE 6:**
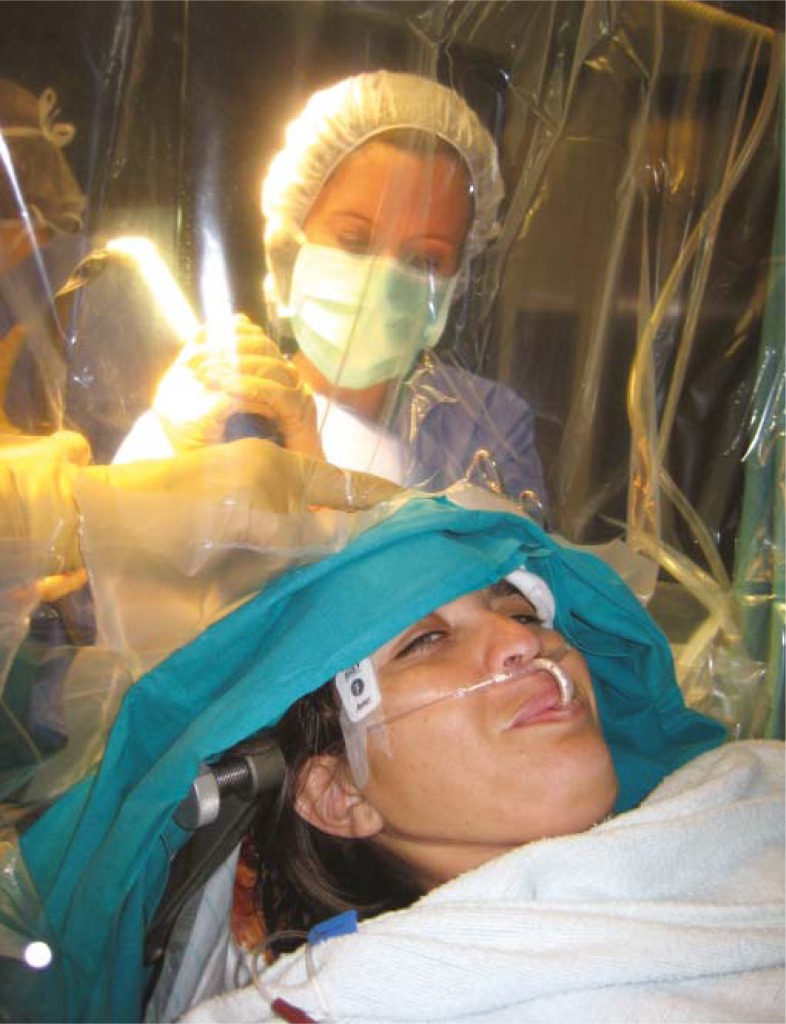
Awake craniotomy on a patient with malignant glioma.

**FIGURE 7:**
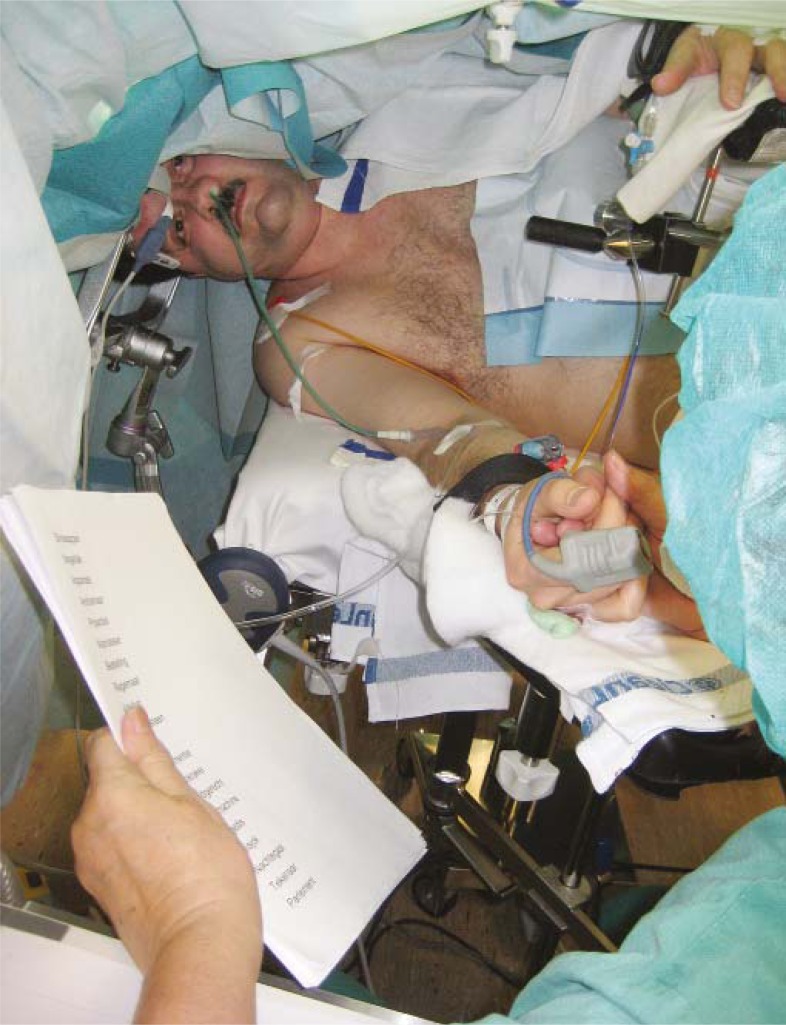
Surgery for a malignant glioma on awake patient.

In all cases where real-time monitoring of higher neurological functions is wishful, awake craniotomy has found its place. Awake surgery is a variant of the direct cortical stimulation. It is used mainly to determine speech areas of the brain, however motor and sensory areas can also be localized. It remains a challenge for the anaesthesiologist to guide the patient in a stable and comfortable way through the procedure while keeping him awake for the sufficient interaction. Local anaesthetics play an important role in the awake surgery. During craniotomy and dural incision, a mild sedation without intubation is usually used. During the tumour removal, both the neurosurgeon and the neurophysiologist communicate with the patient to detect any speech disturbances while the speech area is stimulated by means of the direct cortical stimulation (Figures 6,7).

## Neuroendoscopy

Neuroendoscopy can provide a minimally invasive approach for biopsy or resection of the tumour, as well as the management of concurrent obstructive hydrocephalus.^10^,^12^ Neuroendoscopic approaches use the natural CSF-filled ventricular cavities in the brain as a conduit for accessing tumours. Illumination from the tip of the neuroendoscope allows the neurosurgeon to navigate within the ventricular system and optimally visualize the tumour. The neuroendoscope can be used to perform resections of intraventricular and paraventricular tumours involving deep midline portions of the brain. The direct visualization of tumours can allow for more accurate tissue sampling and improved haemostasis. In addition, the ability to simultaneously manage tumour-related hydrocephalus through a third ventriculostomy can prevent the placement of ventriculo-peritoneal shunts. The approach to the ventricular system is usually through the right frontal lobe. Because the diameter of the neuroendoscope is less than 1 cm, the exposure and trajectory to the CNS lesion is achieved in a minimally invasive fashion. Most skin incisions measure approximately 2–3 cm in length, and the opening in the skull is about 1–2 cm. No significant brain retraction is needed. Neuronavigation technology allows the surgeon to properly correlate the location of the endoscope with MRI of the brain.

## Stereotactic needle biopsy

**FIGURE 8:**
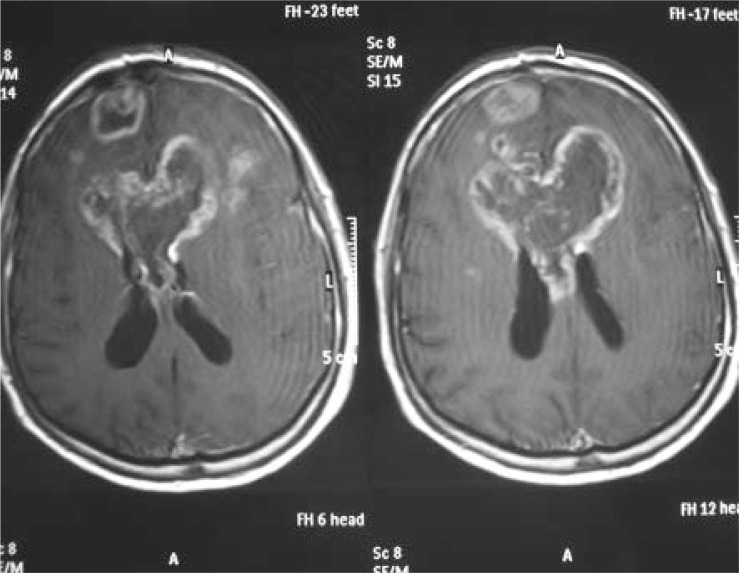
Nonresectable glioblastoma of the corpus callosum.

A needle biopsy should be offered to all patients with nonresectable gliomas (Figure 8) to allow for the histology-guided adjuvant therapy.^13^ Even with resectable tumours, prior needle biopsy is sometimes preferred to the immediate tumour resection in order to allow for the more individualized approach. While frameless neuronavigation–guided needle biopsy is more comfortable for the patient and easier to perform, several centres opt for more accurate stereotactic needle biopsy. *Stereotactic frame* is based on an imaginary 3D Cartesian coordinate system and is also used in stereotactic radiosurgery and deep brain stimulation.

## Brachytherapy

BCNU wafers or iodine-125 seed implants are indicated as an adjunct to surgery in patients with newly-diagnosed or recurrent high-grade gliomas after the microsurgical resection.^14^,^15^ Increasing body of data suggests that the combination of BCNU implants within the multimodal treatment strategy may provide a prolonged survival in patients with glioblastoma.^14^.^16^ Known surgical complications include convulsions, intracranial infections, abnormal wound healing and brain oedema.^16^ The communication between the surgical resection cavity and the ventricular system should be avoided to prevent implants from migrating.

## Nanotecnology

Although not yet used in neurosurgical practice, it is expected that within the next 10 years, nanotechnology will furtherly advance the surgical management of malignant gliomas.^10^ The multifunctional clinical nature of nanotechnology will provide for the targeting, imaging, and therapy of infiltrating brain tumour cells that escape the surgical treatment. Therapeutic nanoparticles coated with various drugs and conjugated to brain tumour-specific antibodies will be delivered systemically or locally to brain tumours. The ability to image nanoparticles by conventional methods, such as MRI, will provide precise information regarding therapeutic agent delivery and therapeutic follow-up. The local hyperthermia treatment of malignant gliomas might also be possible with nanoparticles using alternating magnetic fields that are safe for non-cancerous cells.^10^ However, the surgical resection will still be required to debulk malignant brain tumours and alleviate the mass effect on the surrounding brain.

## Conclusions

Although the recent prolongation of the survival of patients with malignant brain tumours is primarily attributed to chemo- and radiotherapy, the surgical intervention remains crucial. The use of several new techniques in brain surgery can facilitate the extensive resection of these tumours and make it safer for the patient.

## Figures and Tables

**FIGURE 2A:**
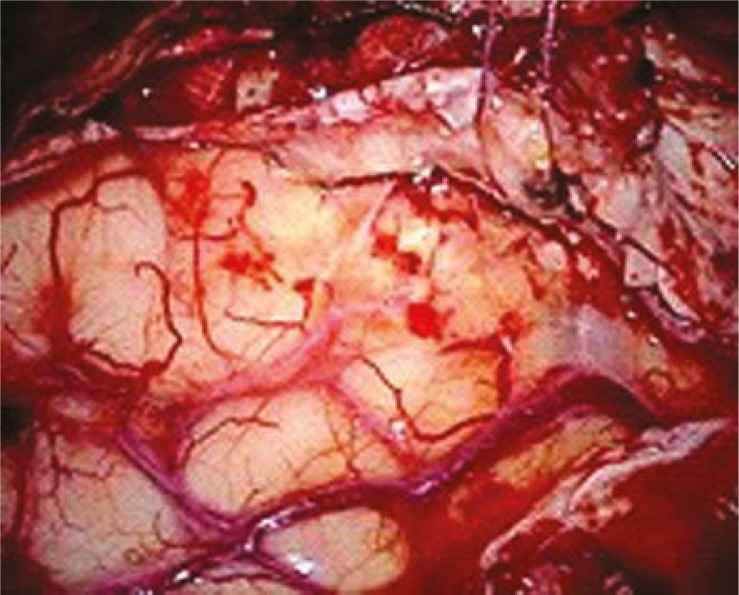
Resection of a malignant glioma - an intraoperative microscopic view of the tumour resection cavity (white light).

**FIGURE 2B:**
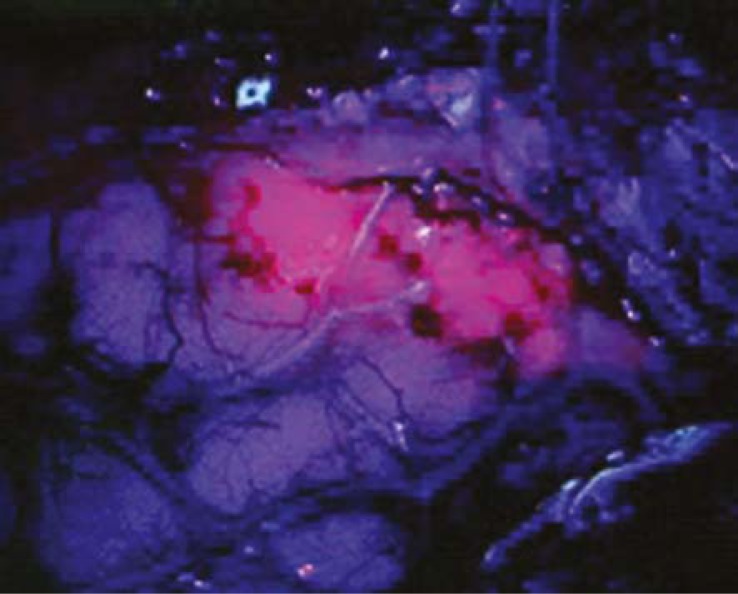
Resection of a malignant glioma using intraoperative fluorescence - an intraoperative microscopic view (blue light).

**TABLE 1:** Different techniques facilitating malignant glioma surgery

	**preoperative**	**intraoperative**
imaging	CT MRI angiography 3D planning PET CT	neuronavigation intraoperative ultrasound fluorescence–guided resection intraoperative MRI
functional	fiber tracking functional MRI transcranial magnetic stimulation	direct cortical stimulation awake surgery
